# Retinopathy among Adult Diabetics and Its Predictors in Northwest Ethiopia

**DOI:** 10.1155/2022/1362144

**Published:** 2022-02-15

**Authors:** Mulualem Birhan Takele, Dube Jara Boneya, Hailemariam Abiy Alemu, Tesfa Birlew Tsegaye, Molla Yigzaw Birhanu, Simegn Alemu, Tsige Gebre Anto

**Affiliations:** ^1^Simada Health Center, Simada District, South Gondar Zone, Amhara Regional State, Ethiopia; ^2^Department of Public Health, College of Health Sciences, Debre Markos University, Debre Markos, Ethiopia PO. Box 269

## Abstract

**Background:**

Diabetic retinopathy is the leading cause of blindness among working-aged adults worldwide, including developing countries such as Ethiopia, and the burden of diabetes-related blindness is undeniably posing a massive challenge to the health care system. Diabetes and its micro- and macrovascular complications are becoming more prevalent among Ethiopian diabetics. For that reason, the purpose of this study was to assess the incidence of diabetic retinopathy and its predictors among diabetics in Ethiopia.

**Methods:**

A hospital-based retrospective cohort study was conducted using 494 randomly selected diabetics aged above 18 years at Felege Hiwot Comprehensive Specialized Hospital from 2011 through 2014 and was followed until December 2019. The preliminary and longitudinal data was abstracted into demographics, clinical, and physiological attributes using a standardized structured questionnaire. The collected data was entered into the system using EpiData version 4.2 and analyzed using STATA version 14.0. The survival experience of the patients was assessed using the Kaplan-Meier survivor function. The predictors of diabetic retinopathy were identified by the Cox proportional hazard model. Bivariable and multivariable Cox proportional hazard models were computed, and variables having a *P* value of < 0.05 in the multivariable Cox proportional hazard model were declared as significant predictors of diabetic retinopathy.

**Results:**

During the follow-up, the overall incidence rate of diabetic retinopathy was 48 per 1000 person-years (95% CI: 40.0–57.0). Age in years (AHR 1.02; 95% CI: 1.00-1.04), fasting blood sugar level (AHR 1.02; 1.00-1.04), hypertension (AHR 2.61; 95% CI: 1.47-4.63), DM patients who had LDL > 100 mg/dl (AHR 2.73; 95% CI: 1.32-5.64), total cholesterol > 200 mg/dl (AHR 2.22; 95% CI: 1.08-4.55), and positive proteinuria (AHR 1.74; 95% CI: 1.10 -2.73) were found to be the significant predictors of diabetic retinopathy.

**Conclusion:**

The overall incidence rate of diabetic retinopathy was found to be high in both type 1 and type 2 DM. Age, fasting blood sugar levels, hypertension, proteinuria, dyslipidemia, and high systolic blood pressure were all predictors of the development of diabetic retinopathy. Controlling glycemia, dyslipidemia, proteinuria, and blood pressure is critical for halting the progression of diabetic retinopathy.

## 1. Introduction

Diabetes mellitus (DM) is a metabolic disease that affects the metabolism of carbohydrates, fat, and protein and is caused by the loss of insulin-producing cells in the pancreas or decreased tissue sensitivity to insulin, resulting in an increased level of glucose in the blood and high mortality associated with its complications, such as diabetes-related retinal damage [1, 2].

Diabetic retinopathy (DR) is a potentially blinding ocular complication of diabetes [3], and it is one of the microcirculatory lesions of the general diabetic disease, which can be caused by lesions in the vessel walls, changes in blood flow, or platelet changes [4]. The main stages of DR are three (early and severe nonproliferative, proliferative DR, and diabetic macular edema) [4]. From a public health perspective, early detection and secondary intervention are essential, because vision loss resulting from DR can usually be prevented with timely and effective treatment. DR is one of the most challenging problems facing ophthalmological research. Today, it is one of the most frequent causes of blindness, since its incidence has increased all over the world [5–8].

The International Diabetic Federation estimated that the global prevalence of DR for the period 2015-2019 was more than 25%. The lowest prevalence was in Europe at around 20% and in South East Asia at 10% and the highest in the Middle East, North Africa, and the Western Pacific region at 40% [9]. A report in rural China showed that more than two-fifths of diabetic had DR [10]. In African countries, the prevalence of patients with diabetes who have DR is approximately one-third [11]. A systematic study of 21 African countries found that the DR had 30.2-31.6% [11]. The overall prevalence of retinopathy was 19.48% in Ethiopia [5]. The incidence of DR was 18.57% in Tikur Anbessa Referral Hospital [12] and 35.96 cases per 1000 persons per year at Arba Minch General Hospital in Ethiopia. The incidence and risk factors of diabetic retinopathy in developed countries have been well documented. However, data on incidence and predictors is scarce in Ethiopia [13].

DR is the leading cause of blindness among productive age groups around the world [3]. Approximately one-third of people with diabetes develop some degree of DR, and it has become the leading cause of vision loss and blindness in adults [7]. The World Health Organization (WHO) estimates that DR accounts for 4.8% of the 37 million cases of blindness worldwide [14]. In South Africa, DR is the 4th leading cause of blindness, after cataract, glaucoma, and age-related macular degeneration, and the 3rd leading cause of severe visual impairment, after refractive error and cataract in persons aged ≥ 50 years [15]. Prevention of visual impairment in DR is achieved principally through control of diabetes, early detection of retinal changes, and timely treatment of sight-threatening lesions of the retina [16, 17]. Screening of people with diabetes is a cost-effective way to prevent DR [18, 19].

Diabetes duration is a major risk factor for developing DR [20]. Almost all people with type 1 diabetes and more than 60% of those with type 2 diabetes are at risk of developing DR within the first 20 years of being diagnosed [4]. The median DR duration was 7.9 years in the world [7]. In Europe, 50% of type 1 DM with no DR at baseline have been shown to develop retinopathy in 5-7 years [21]. The median survival time for DR diagnosis was 58 months in Iran [22]. DR was recorded in 42% and 84.7% of diabetic patients after 5 and 10 years of follow-up in England, respectively [23]. A United Kingdom prospective diabetic study showed that in a 6-year follow-up study, DR was observed in 41% of all diabetics [24]. In Israel, during a mean follow-up of 4.5 years, the cumulative incidence of DR was 18.8% [25]. In a study conducted at Tikur Anbessa Referral Hospital [12], the median survival time was 74.07 months, whereas a 10-year study was conducted at Arba Minch General Hospital in Ethiopia [13].

The most common risk factors related to DR are sociodemographic characteristics such as age, sex, body mass index (BMI), hypertension, poor glycemic control, type 2 diabetes mellitus, high blood pressure, cholesterol level, and time since diabetes diagnosis. The importance of the above factors, however, varies between studies [5, 7, 12, 26, 27]. Ethiopia has been lacking in basic preventive and curative medicines, technologies, and procedures in primary health care for DM. At the national level, there are no standard criteria for referral of patients from primary care to higher levels of care [6]. Evidence of time to DR on its incidence and predictors among diabetics in Ethiopia are scarce. This study is aimed at determining time to DR and predictors in adult diabetics in Felege Hiwot Comprehensive Specialized Hospital, Ethiopia.

## 2. Patients and Methods

### 2.1. Study Setting, Period, and Design

A hospital-based retrospective cohort study design was conducted in Felege Hiwot Comprehensive Specialized Hospital (FHCSH), Bahir Dar, Ethiopia, from 28 February 2020 through 21 March 2020. FHCSH is located at 565 km away from Addis Ababa, the capital city of Ethiopia. FHCSH is a tertiary health care-level hospital serving for 12 million population of the catchment area of northwest Ethiopia. There were around 1,150 diabetics who had a follow-up per month in the hospital.

### 2.2. Population

The source population of FHCSH was all diabetics over the age of 18. All newly diagnosed type 1 or type 2 diabetics aged above 18 years between 2011 and 2014 were the study population. All diabetics aged above 18 years and who had a follow-up for treatment between 2011 and 2014 were included in the study. Patients with gestational diabetes and diabetes and retinopathy at the same time were excluded from the study.

### 2.3. Sample Size Determination and Sampling Procedure

The minimum calculated sample size for this study (553) was determined via Cox's proportional hazard with the assumption of sample size for a test of *H*0: *β*1 = 0 versus *Ha*: *β*1 ≠ 0 of a binary covariate in a Cox proportional hazard model given an alternative coefficient *b*_1_. Under the assumption of proportional hazards, log (*λ*2 (*t*)/*λ*1 (*t*)) = *b*_1_ is a constant. Assuming that *H*_1_ (*t*) = *H*_2_ (*t*) and *d* is the cumulative probability of observing an event, the two-sided sample size formula with significance level *α* and power 1 − *β* is given by

(1) $$\begin{array}{c} n=\frac{\left(Za/2+Z\;\beta \right)\;2}{\text{b}2p1p2d} \end{array}$$

using sample size for clinical research formula [3], where *n* is the sample size, *z*_*a*/2_ is the significant level of *α* at 5%, Z_*β*_ (power) is 80%, *p*1 is the standard deviation of the covariates, and *b* is the coefficient of each covariate. Sex (male), diabetic type (type 2), creatinine level, and triglyceride level above 150 mg/dl were covariates (source) used to calculate the sample size; the values (*b*-coefficients) were 0.662, 1.389, 0.577, and 0.952, respectively.

### 2.4. Variables

The primary outcome of this study was time to DR and diabetic retinopathy diagnosis. The diagnosis that included medical history, ophthalmic examination, screening with retinal photographs, and regular follow-up were the independent variables. DR diagnosed with dilated eye examination using tonometry and ophthalmologic instruments. The first characteristics evaluated were sociodemographic factors such as age, gender, and place of residence. The second characteristic measured contained clinical components of HTN, BMI, DM type, family history of DM, and the type of DM treatment. The third characteristic considered was the physiologic component comprising FBS, SBP, DBP, HDL-C, LDL-C, triglyceride level, total cholesterol level, creatinine, and proteinuria. HDL-C, LDL-C, triglyceride, and total cholesterol were categorized as high and low based on the National Cholesterol Education Program 3 (NCEP-III) and WHO guidelines.

### 2.5. Operational Definitions

DR was defined by both direct and indirect ophthalmoscopy assessments done by retinal specialists confirmed by fundus photography. Diabetic retinopathy was defined as a microvascular complication of diabetes that was evaluated by clinical examination or indirect ophthalmoscopy by ophthalmologists and classified as present (yes) or absent (no) from the charts based on ophthalmologists decision [12]. Time to DR was the time gap in years between diagnoses of diabetes mellitus and first episode of DR [4].

The primary outcome of this study was time to diabetic retinopathy. However, it was considered as censored when diabetic patients did not develop DR throughout the study period, lost to follow-up, died before the onset of DR, and transferred out.

Survival time was the time from the diagnosis of diabetic mellitus to development of DR or to censor in a year; censored was considered when diabetic patients had loss to follow-up, transferred out, died before the onset of DR, and did not develop DR throughout the study period. Hypertension systolic and diastolic blood pressure of individual ≥ 140 and ≥90 mmHg, respectively, at baseline or on antihypertensive medication [5].

Dyslipidemia was diagnosed if total cholesterol > 200 mg/dl, LDL − cholesterol > 100 mg/dl, HDL − cholesterol < 40 mg/dl, and triacylglycerol > 150 mg/dl or on medication of dyslipidemia [6, 7].

For high blood glucose levels, fasting plasma glucose levels are ≥126 mg/dl.

Proteinuria was defined as positive if the urine albumin concentration is more than 30 mg/mmol and negative if it is <30 mg/mmol.

BMI was classified as underweight (<18.5 kg/m^2^), normal (18.5 kg/m^2^-24.9 kg/m^2^), and overweight (>25 kg/m^2^).

### 2.6. Data Collection Tool, Procedures, and Quality Assurance

A structured data extraction checklist adapted from previous studies was used to extract all necessary information from patients' medical registrations [12]. There are two Bachelor of Science nurses' abstract data under the supervision of a trained supervisor. Patients who were diagnosed with diabetes and retinopathy at the time of diagnosis and patients' charts with missed key variables were excluded from the study. The reviewed records were identified by their medical registration or card numbers. Patients with missing key predictor variables at baseline, including age, diabetic type, and creatinine and hypertension status, were excluded from the study. With a uniform checklist, relevant secondary data was extracted from the patient's intake form, follow-up card, DM registration book, and electronic information databases. To ensure the quality of the data, the principal investigator provided one-day data collection training to data extractors and supervisors prior to data abstraction on how to extract data from individual charts. Data completeness was assessed daily by the principal investigators. Furthermore, the pretest was conducted using 5% of the sampling charts outside the study area.

### 2.7. Data Management and Analysis

The extracted data was entered into a computer package through EpiData version 4.2, then exported to the STATA software version 14.0 for cleaning, coding, categorizing, merging, and checking completeness and consistency, and outliers were summarized and for further analysis accordingly.

The percentage and frequency of patients regarding all covariates were summarized using descriptive statistics. The survival experience of the patients was assessed using the Kaplan-Meier survival function. The log-rank test was used to compare the survival experiences of the various groups of subjects using categorical variables. The independent predictors of DR were estimated via the Cox proportional hazard model. Interactions were checked, and cofounders were controlled through multivariable analysis. The model was built through a stepwise backward elimination procedure, and the goodness of fit of the model was assessed by using the Cox-Snell residual technique. The Schoenfeld residual test, the interaction of each covariate with time and graphical methods, was used to check the Cox proportional hazard (PH) assumption and fulfilled the assumption. The potential candidate predictors in the full model were selected by bivariable Cox proportional hazard regression analysis with a cut-off point *P* value ≤ 0.25. The multicollinearity of variables in the final fitted model was checked using the variance inflation factor (VIF) with a cut-off point mean VIF > 10; the mean value of the VIF was found to be 1.69. The incidence rate was computed for the entire cohort by dividing the total number of incident cases of DR by the total person-years of follow-up. The association between predictors and the hazard of DR was determined using an adjusted hazard ratio (AHR). Finally, variables with a *P* value of < 0.05 in the multivariable Cox proportional hazard regression analysis were considered to identify significant predictors for diabetic retinopathy.

## 3. Result

### 3.1. Baseline Characteristics

Among the 553 diabetic patients' chart, 494 (89.33%) records were included in the final analysis. The median age of the diabetic patients at the time of diabetic diagnosis was 37 years with IQR (30–48 years). From the 494 study participants, 296 (59.2%) were belonged to the age group 30 to 60 years, and 278 (56.28%) and 261 (52.83%) were male and rural residents, respectively. Among the 494 diabetic records, 169 (34.21%) and 280 (56.68%) were hypertensive and type 2 diabetic participants, respectively. The study findings revealed that 115 (23.28%) participants had a family history of diabetes. The mean BMI of study participants was 22.9 kg/m^2^ (SD ± 2.1). More than half of the study participants, 261 (52.83%), were taking oral drug regimens for diabetes treatment.

Median fasting blood sugar was 299 mg/dl with IQR (238–364). Almost three-fourth participants, 363 (73.48%), had negative proteinuria. Mean systolic and diastolic blood pressure was 123 mmHg (SD ± 1.025) and 78.33 mmHg (SD ± 0.56), respectively. Majority of diabetic patients, 276 (55.78%), had more than 100 mg/dl low-density lipoprotein. Majority 80% (398) and 73% (347) of the study participants presented with high-density lipoprotein greater than 40 mg/dl and total cholesterol less than 200 mg/dl, respectively, whereas the median creatinine was 0.7 mg/dl IQR (0.56–0.83) (Table 1).

### 3.2. Incidence and Time to Diabetic Retinopathy

**Figure 1:**
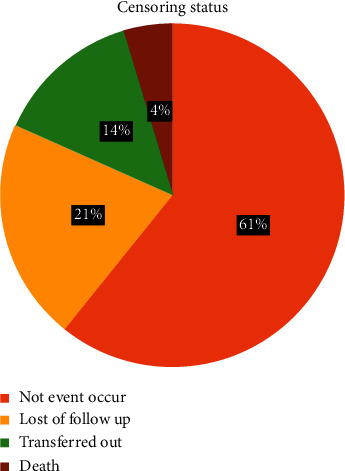
Censoring status of diabetic patients in Felege Hiwot Comprehensive Specialized Hospital, Ethiopia, 2020.

Four hundred ninety-four study participants were followed for a minimum of 1.01 and a maximum of 8.87 years after their initial DM diagnosis with 6.45 years IQR (4.74–7.49) median follow-up time and 2959.31 years total follow-up time with median survival time 8.7 years IQR (6.53–8.86) and 0.048 incidence rate. Among those, 142 (28.74%, CI: 25.91%–32.90%) had diabetic retinopathy. From 494 study participants, 352 were censored. Among the 352 censored participants, 214 (60.80%) did not develop diabetic retinopathy, and 74 (21.02%) were lost to follow-up, 48 (13.64%) were transferred to other health facilities, and 16 (4.55%) died (Figure 1).

The median time for free of diabetic retinopathy among diabetic patients was 8.7 years with IQR (6.53–8.86). The overall incidence rate in this follow-up was found to be 48/1000 person-year (P-Y) observations. The incidence rate of diabetic retinopathy in the first three-year follow-up was 14/1000 person-years with 1443.67 person-years at risk. High incidence rate of diabetic retinopathy was observed after six-year follow-up (131/1000 person-years) with a 372.9 person-years at risk (Table 2).

The cumulative survival rates of diabetic retinopathy at the end of the first year, at the end of the fifth year, and at the end of the eighth year were 0.98, 0.79, and 0.46, respectively.

**Figure 2:**
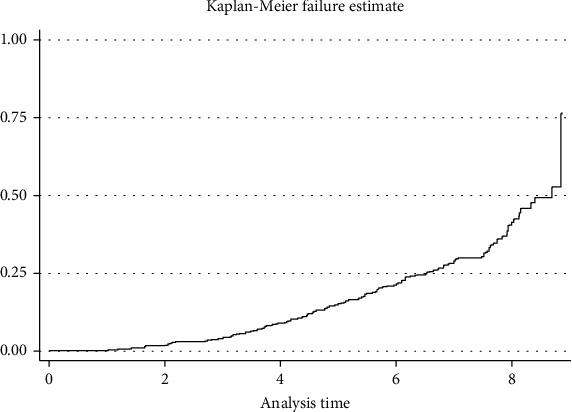
Kaplan-Meier failure estimates of diabetic retinopathy in Felege Hiwot Comprehensive Specialized Hospital, Ethiopia, 2020.

Even if there was no statistically significant difference, the incidence rate was 29/1000 person-year (P-Y) observations and 63/1000 person-year (P-Y) observations among type 1 and type 2 diabetic patients, respectively. The incidence rate of diabetic retinopathy among male and female diabetic patients was 49/1000 person-years and 48/1000 person-years. The incidence rate of diabetic retinopathy among the age group less than 30 years, between 30 and 60 years, and greater than 60 years was 2/1000, 58/1000, and 170/1000 person-years (P-Y). The incidence rate of diabetic retinopathy was high in those hypertensive diabetic patients (123/1000 person-year observation) compared to nonhypertensive diabetic patients (10/1000 person-years) (Figure 2).

### 3.3. Cox Proportional Hazard Assumption

Cox proportional hazard assumptions were assessed with Schoenfeld residual test, graphical method, and interaction of each covariate with time. In the global test, the probability of chi-square was computed and gave 0.17. The interaction of each covariates with time was assessed. No covariates were statically interacted with time. Therefore, Cox proportional hazard regression analysis model was computed for analysis.

### 3.4. Predictors of Time to DR among Diabetic

Bivariable Cox proportional hazard regression analysis was conducted to select potential candidate variables for the multivariable analysis selected using the preset *P* value criteria cut-off point ≤ 0.25. Accordingly, except sex and residence, other predictors satisfied the criteria and were a potential candidate for the multivariable analysis.

In multivariable Cox proportional hazard regression analysis, baseline age, hypertension status at baseline, baseline fasting plasma glucose level, baseline proteinuria, baseline LDL level, baseline cholesterol level, and baseline triglyceride level have statistically significant association with the development of DR.

This study indicated that the age of diabetic patients was found to be predictors of the incidence of diabetic retinopathy in which the risk of DR is increased by 1.02 (AHR 1.02, CI: 1.01–1.04, *P* < 0.008) times as the age increased by year at any given time by holding other predictors constant. The risk of DR is increased by 2.6 (AHR 2.61, CI: 1.47–4.63, *P* < 0.001) times at any given time among those who are hypertensive at baseline compared to nonhypertensive at baseline by keeping other predictors constant. The risk of DR was increased by 0.2% (AHR 1.002, CI: 1.001–1.004, *P* < 0.007) as baseline fasting plasma glucose increased by one mg/dl at any given time. Diabetic patients with positive baseline proteinuria had a risk of DR by 1.74 (AHR 1.74, CI: 1.10–2.73, *P* < 0.016) times at any given time compared to diabetic patients with positive baseline proteinuria by keeping other predictors constant.

The risk of DR increased by 2.73 (AHR 2.73, CI: 1.32–5.64, *P* < 0.007) times at any given time among DM patients with LDL greater than 100 mg/dl compared to LDL less than 100 mg/dl at baseline by keeping other predictors constant. The risk of DR who had a triglyceride at baseline greater than 150 mg/dl was increased by 2.55 (AHR 2.549, CI: 1.19–5.46, *P* < 0.016) times at any given time compared to those who had triglycerides at baseline less than 150 mg/dl by holding other predictors constant. Total cholesterol greater than 200 mg/dl at baseline was found to be a risk of DR in which the risk is increased by 2.22 (AHR 2.22, CI: 1.08–4.55, *P* < 0.029) times at any given time compared to diabetic patients with less than 200 mg/dl by holding other predictors constant. The risk of DR is increased by 1.68 (AHR 1.68, CI: 1.14–2.5, *P* < 0.01) times at any given time, for those who had systolic blood pressure greater than 140 mmHg compared to those who had systolic blood pressure less than 140 mmHg by keeping other predictors constant (Table 3).

### 3.5. Model Adequacy

**Figure 3:**
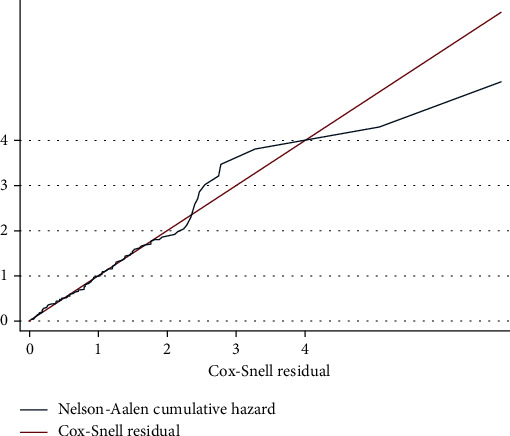
Model adequacy for time to retinopathy and its predictors among adult diabetic patients in Felege Hiwot Comprehensive Specialized Hospital, Ethiopia, 2020.

After fitting the Cox proportional hazard model, the adequacy of the model was checked by Nelson-Aalen cumulative hazard with Cox-Snell residuals. The graph suggests that the hazard function follows the line very closely except for very large values of time. It is widespread for models with censored data to have some wiggling at large values of time [8]. Overall, this would conclude that the final model was well adequate (Figure 3).

## 4. Discussion

In this study, the incidence of diabetic retinopathy was 48 persons/1000 person-years higher than the study conducted at Jimma University Medical Center with 36.9 person/1000 person-years [9], at Tikur Anbessa Teaching Hospital (TATH) with 2.65 per 1000 person-year observation (95% CI: 2.54-4.05) [10], and 36 cases per 1000 patients per year at Arba Minch General Hospital [10]. The possible reason may be followed up years. The incidence of diabetic retinopathy in this study is lower than the study conducted in Iran and England [11, 12]. The possible reason might be follow-up years and health care system and screening programs. This incidence was consistent with a study conducted in South Africa, 50 cases per 1000 person-years in type 1 and 47 cases per 1000 person-years in type 2 diabetic patients [13]. The study showed that the retinopathy rate was 28.74% in 9-year follow-up. This finding is similar to study conducted in Israel [14].

The median survival time of diabetic retinopathy in this study was higher than in the study conducted in TATH [4] but lower than the study conducted in Arba Minch General Hospital [10]. The possible reason may be follow-up years. The time free of diabetic retinopathy among DM patients in this study was lower than median survival time of DR in England [11]. This is may be due to the care given to diabetic patients. The median survival time for diabetic retinopathy in this study is higher than the study conducted in Barbados and the United States [15, 16]. A possible reason may be a screening program for DR after DM diagnosis. In this study, the median survival time of DR was consistent with a study conducted in Sweden [17].

High-density lipoprotein and family history of diabetic patients were not statistically associated with DR development. This is supported by a study conducted in TATH [4]. There was a high incidence of diabetic retinopathy among type 2 diabetic patients in this study, but there was no statistically significant difference which was supported by a study done in South Africa [13]. Many studies reported that type 2 diabetes mellitus was an independent statistically significant predictor of diabetic retinopathy [4]. In contrast, type 1 diabetes mellitus was a significant risk factor for diabetic retinopathy by a study done in Spain [18] and the United States [15].

This study indicated that the age of participants at the time of diabetic diagnosis is the risk of diabetic retinopathy in which the risk of DR is increased by 1.02 times as a year increment that is statistically significant in lining with a study conducted in Arba Minch General Hospital [10]. Many studies agreed that age at the time of diabetes mellitus diagnosis was a significant predictor for diabetic retinopathy development [4, 10, 11, 19], but a study in Israel showed that age was not a significant risk factor for diabetic retinopathy [14].

A Cox regression analysis showed that the time to retinopathy was significantly shortened in those hypertensive diabetic patients. This is lined with a study conducted in England [11]. Hypertension was a particularly significant risk factor for diabetic retinopathy in Oklahoma Indians [20]. Studies done in Oman recorded that the retinopathy rate was higher in patients with hypertension [21].

The risk of diabetic retinopathy is increased by fasting blood sugar increase lined with a study conducted at AMGH [10]. Elevated baseline fasting blood glucose level predicted retinopathy incidence indicated by Blue Mountain Eye study [22]. Many studies investigated that poor glycemic control is the main predictor for diabetic retinopathy among adult diabetic patients [12, 14, 18]. The development of retinopathy (incidence) was strongly associated with baseline glycemia in the United Kingdom [23].

The study identified that among DM, patients with positive proteinuria had a shorter time than DM patients with negative proteinuria to develop diabetic retinopathy. This result is lined with that of a study conducted in Iran [12]. Contrarily, the study conducted by Sasso et al. showed us that no difference in albumin excretion rate (AER) was observed between presence and absence of DR. This may be due to the difference in study design and the specificity of the study [24].

The survival time of diabetic retinopathy is shortened by high levels of low-density lipoprotein, high levels of total cholesterol, and high triglyceride levels. This study is in lined with study in TATH [4] and with the study conducted in Italy [25].

Low-density lipoprotein remains an independent risk factor for diabetic retinopathy, but no other lipid variables (HDL cholesterol or triglycerides) were significant in the survival analysis in Spain [18] and the United Kingdom [23]. Lipid studies often create controversy, such as Yau et al. meta-analysis [26], which reported that higher total cholesterol, LDL, and triglycerides were linked to retinopathy, and similar data were reported in the University of Gondar Referral Hospital among type 2 diabetic patients [27].

As systolic blood pressure elevated, the survival time of diabetic retinopathy shortened. This finding is comparable with a study done in Barbados and Iran [12, 16]. The development of diabetic retinopathy (incidence) was strongly associated with baseline high blood pressure in the United Kingdom and Oklahoma Indians [20, 23]. On the other hand, systolic blood pressure was not a significant risk factor for diabetic retinopathy among adult diabetic patients in some studies [4]. There was no impact of blood pressure on retinopathy development within the study in German among type 1 diabetic patients [28].

## 5. Limitations of the Study

Despite its strength, the current study has some limitations. Because of the retrospective nature of the study, the lack of full records on factors like drug adherence, physical exercise, smoking, and dietary patterns may underestimate the effects and subject variations in the development of the incidence of DR. Even though the study was conducted at the largest national medical center in the country for DM follow-up, the findings might not be representative of the diabetes population in the country because of a possible selection bias.

## 6. Conclusion

In general, DR among patients with type 1 and type 2 DM accounts for 29 and 63 per 1000 P-Years, respectively. Even though the incidence of DR is becoming a public health burden in the country and varies across all DM types, the incidence of DR was not statically different between type 1 and type 2 DM in the current study. The median survival time of DR among newly diagnosed diabetic patients was short, and the overall incidence rate in this follow-up was found to be high. Age at diabetes diagnosis, fasting blood sugar level, hypertensive status, proteinuria, LDL, triglyceride, and total cholesterol level at baseline independently predict an increased diabetic retinopathy development/decreased survival time of DR among both diabetic groups. Therefore, the results of this study suggest that control of glycaemia, dyslipidemia, and blood pressure is important in the prevention of DR. Special care should be given to those diabetic patients with hypertension and who had a high levels of LDL, triglyceride, and total cholesterol. We recommend further rigorous studies using primary data, which includes variables that were unavailable from the medical charts.

## Figures and Tables

**Table 1:** Baseline sociodemographic characteristics of diabetic adults in Felege Hiwot Comprehensive Specialized Hospital, 2020 (*N* = 494).

Variables	Categories	Frequency		
		Event	Censored	Total *N* (%)
Sex	Male	79	199	278 (56.28)
	Female	63	153	216 (43.72)
Residence	Urban	72	161	233 (47.17)
	Rural	70	191	261 (52.83)
Hypertension	Yes	121	48	169 (34.21)
	No	21	304	325 (65.79)
Family history of diabetes	Yes	43	72	115 (23.28)
	No	99	280	379 (76.72)
Diabetic type	Type 1	38	176	214 (43.32)
	Type 2	104	176	280 (56.68)
Type of diabetes treatment	Insulin	38	179	217 (43.93)
	Noninsulin	98	163	261 (52.83)
	Mixed	6	10	16 (3.24)
High-density lipoprotein level	<40 mg/dl	63	33	96 (19.43)
	>40 mg/dl	79	319	398 (80.57)
Low-density lipoprotein level	<100 mg/dl	13	291	304 (61.54)
	>100 mg/dl	129	61	190 (38.46)
Triglyceride level	<150 mg/dl	19	319	338 (68.42)
	>150 mg/dl	123	33	156 (31.58)
Total cholesterol level	<200 mg/dl	21	326	347 (73.48)
	>200 mg/dl	121	26	147 (29.76)
Proteinuria	Negative	34	329	363 (74.48)
	Positive	108	23	131 (26.52)

**Table 2:** Overall incidence and incidence rate of DR every three-year interval among diabetic at Felege Hiwot Comprehensive Specialized Hospital, 2020.

Year interval	Person-year at risk	Event	Incidence rate	95% CI
0–3 years	1443.67	20	0.014	0.009–0.021
4–6 years	1142.74	73	0.064	0.051–0.080
Above 6 years	372.9	49	0.131	0.099–0.173
Total	2959.31	142	0.048	0.040–0.057

**Table 3:** Predictors of DR among adult diabetic in Felege Hiwot Comprehensive Specialized Hospital, 2020 (*N* = 494).

Variables	Survival status of categorical predictors		CHR (95% CI)	AHR (95% CI)	*P* value
	Event	Censored			
Age in years	—	—	1.08 (1.06–1.09)	1.02 (1.01–1.04)	0.008
FBS in mg/dl	—	—	1.008 (1.006–1.009)	1.002 (1.001–1.004)	0.007
Hypertension					
No	121	48	1	1	
Yes	21	304	14.69 (9.232–3.40)	2.61 (1.47–4.64)	0.001
Proteinuria					
No	34	329	1	1	
Yes	108	23	12.43 (8.44–18.31)	1.74 (1.10–2.73)	0.016
LDL					
<100 mg/dl	13	291	1	1	
>100 mg/dl	129	61	20.59 (11.6–36.45)	2.73 (1.52–6.65)	0.007
TC					
<200 mg/dl	21	326	1	1	
>200 mg/dl	121	26	22.93 (14.2–36.96)	2.22 (1.08–4.55)	0.029
TG					
<150 mg/dl	19	319	1	1	0.016
>150 mg/dl	123	33	21.88 (13.3–35.99)	2.55 (1.19–5.46)	
SBP					
< 140 mmHg	58	342	1	1	
≥140 mmHg	84	10	7.24 (5.18–10.14)	1.68 (1.14–2.5)	0.01

FBS: fasting blood sugar; LDL: low-density lipoprotein; TC: total cholesterol; TG: triglyceride; SBP: systolic blood pressure.

## Data Availability

The data sets generated during this study are available from the correspondences on reasonable request.

## Abbreviations

AHR: Adjusted hazard rate
BMI: Body mass index
CI: Confidence interval
DBP: Diastolic blood pressure
DM: Diabetic mellitus
DR: Diabetic retinopathy
FBS: Fasting blood sugar
HDL: High-density lipoprotein
HR: Hazard rate
HTN: Hypertension
IDF: International diabetes federation
IQR: Interquartile range
LDL: Low-density lipoprotein
SBP: Systolic blood pressure
TG: Triglyceride
WHO: World Health Organization.
